# Intramuscular myxoma of the deltoid muscle in a swimmer: A rare case report and literature review

**DOI:** 10.1002/ccr3.2483

**Published:** 2019-10-27

**Authors:** Saulius Adamonis, Bassey Enodien, Stephanie Taha‐Mehlitz, Andreas Maurer, Anas Taha

**Affiliations:** ^1^ Clinic for General and Visceral Surgery Spital Einsiedeln Einsiedeln Switzerland; ^2^ Department of General Surgery GZO Spital Wetzikon Wetzikon Switzerland; ^3^ Clarunis Universitäres Bauchzentrum University Hospital Basel Basel Switzerland; ^4^ Department of Orthopedics and Trauma Surgery Rheinfelden Hospital Rheinfelden Switzerland; ^5^ Department of Visceral and Thoracic Surgery Cantonal Hospital of Winterthur Winterthur Switzerland

**Keywords:** benign tumor, deltoid muscle, intramuscular myxoma, myxoma

## Abstract

Intramuscular myxoma (IM) is a rare mesenchymal neoplasm with the incidence of one per 1 000 000 people and is reported greater among women. Our case reports an IM of a deltoid muscle in a young man. Differential diagnosis with sarcoma is important. Surgery is the treatment of choice usually without recurrence.

## Graphical abstract

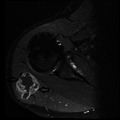

## 1 INTRODUCTION

Intramuscular myxoma (IM) is a rare mesenchymal neoplasm. The current study reports a case of a 34‐year‐old swimmer. Imaging showed an ovoid mass in the deltoid muscle. The differential diagnosis of low‐grade fibromyxoid sarcoma is important when diagnosing IM. Surgical excision is the treatment of choice with a low risk of recurrence.

Myxoma is a benign connective tissue tumor found usually in the heart. The extracardiac manifestations of the tumor range from intramuscular and superficial cutaneous to odontogenic and juxta‐articular myxoma. Thus, intramuscular myxoma (IM) is a type of extracardiac manifestations of myxoma. IM has an incidence of one per 1 000 000 people between the fifth and seventh decades of one's life and occurs more frequently in women with 60% predominance.^1^ Our case report is unique because it is the first deltoid myxoma reported in a man, moreover one younger than 40 years.

The diagnostic imaging options include magnetic resonance imaging (MRI) and ultrasonography, but the definitive diagnosis is histopathological. IM is characterized by bland spindle‐ and/or stellate‐shaped cells embedded in a hypovascular abundant myxoid stroma. The nuclei are small and show no or minimal nuclear atypia.^2^ Histopathologically, S‐100 protein negativity excludes myxoid neurofibromas and low‐grade malignant peripheral nerve sheath tumors, while the absence of vascularity and negativity of the FUS/CREB3L2 translocation decreases the likelihood of sarcoma.^3^

A comprehensive literature review was performed by searching the PubMed‐National Center for Biotechnology Information database, using the keywords “deltoid AND myxoma.” The search yielded 6 case reports published prior to July 2018. Of these, four included cases of solitary myxoma in the deltoid muscle and thus were included in our literature review Table 1. To the best of our knowledge, we report the first known case in English literature of a 34‐year‐old man with myxoma located in the deltoid muscle and aim to discuss intramuscular myxomas in the light of clinical, radiological, and pathological data.

**Table 1:** Intramuscular myxoma of deltoid muscle. Literature overview

Reference	Gender	Age, y	Examination	Size	Biopsy	Treatment
9	F	75	MRI	70 × 40 mm	No	Surgical excision
10	F	44	MRI	34 × 23 × 37 mm	No	Surgical excision
11	F	39	X‐Ray	100 × 90 × 70 mm	No	Surgical excision
12	F	83	MRI	95 × 60 mm	Yes	Conservative, follow‐up after 1 y
Our case report	M	34	MRI	40 × 40 mm	Yes	Surgical excision

Abbreviations: F, female; M, male.

## 2 CASE PRESENTATION

**Figure 1:**
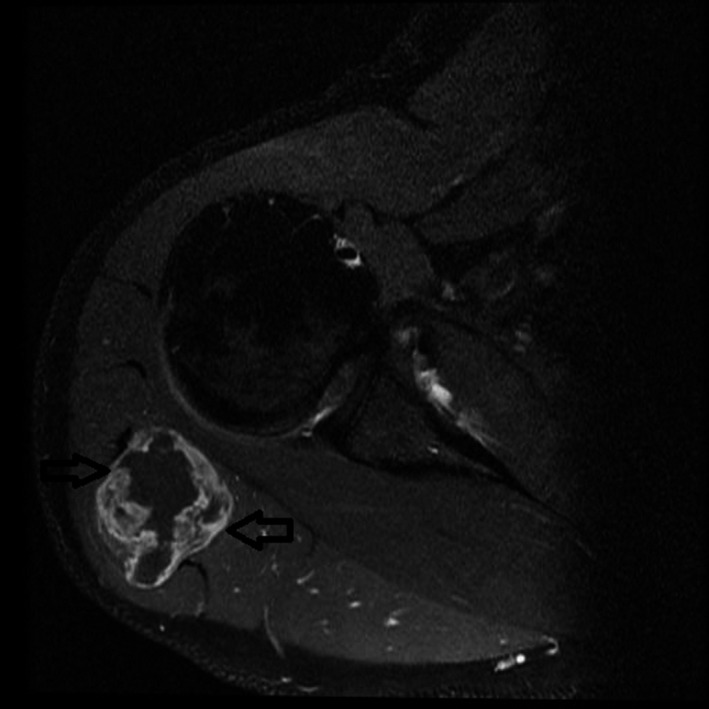
The MRI T2 sequence coronal plane shows polycystic tumor with solid aspects

**Figure 2:**
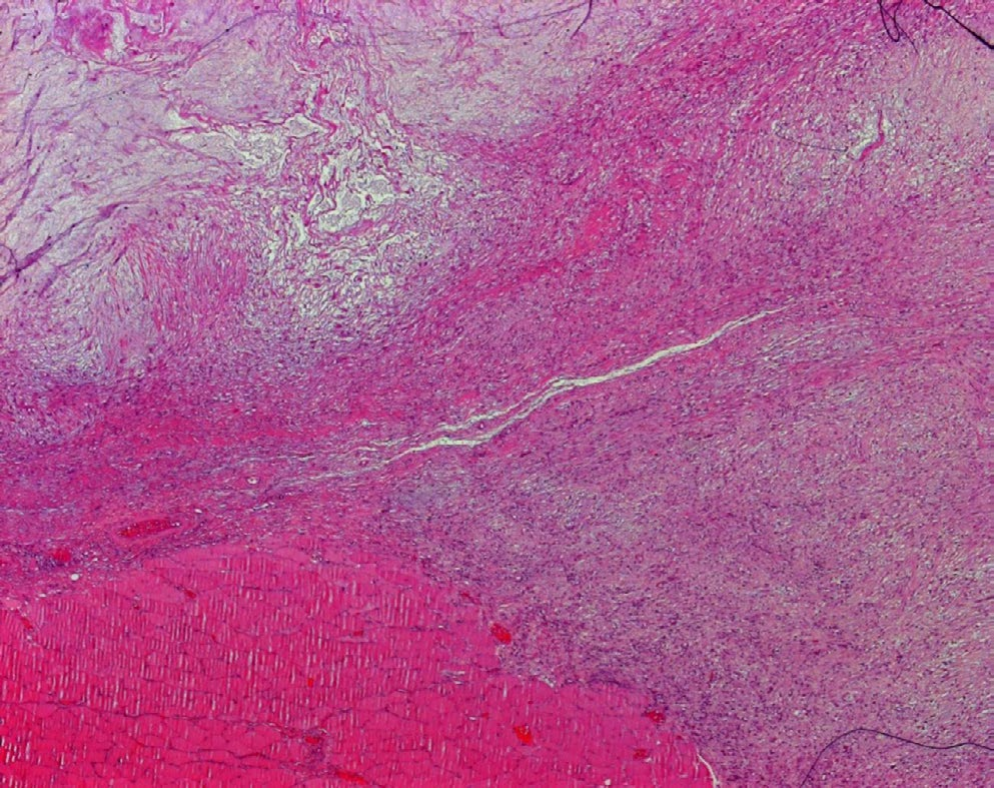
Microscopic appearance with cellular and myxoid areas (hematoxylin‐eosin stain, 5× magnification)

**Figure 3:**
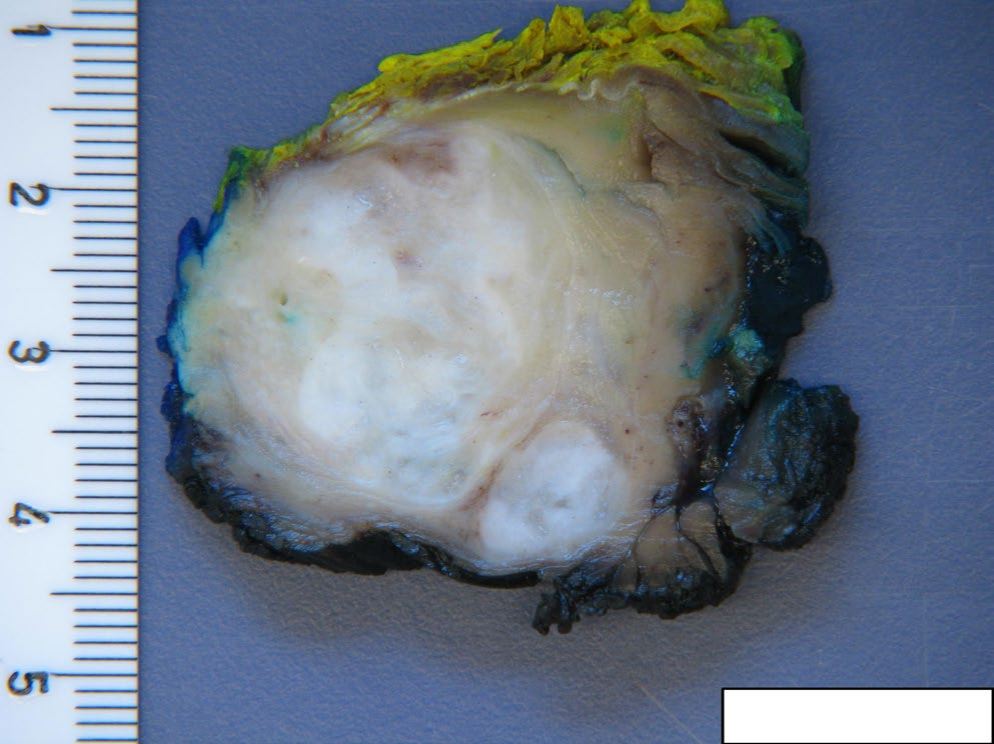
Macroscopic appearance of the intramuscular myxoma

**Figure 4:**
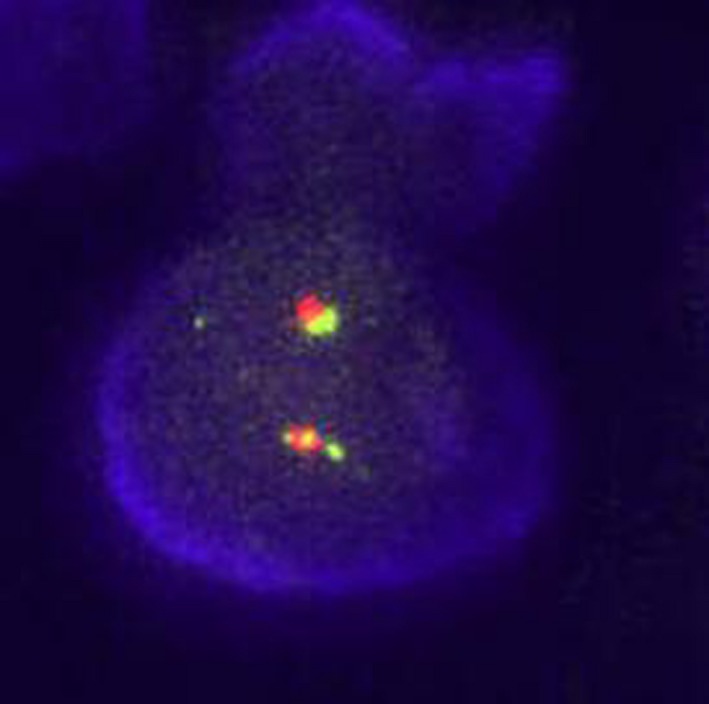
The nuclei of the tumor cells show two fused signals in a fluorescence in situ hybridization (FISH) analysis of the FUS gene, performed with the LSI FUS (16p11) Dual‐Color Break Apart Rearrangement Probe, Vysis. This result confirms the lack of rearrangement of the FUS gene, excluding the translocation

A 34‐year‐old otherwise healthy male swimmer presents with the shoulder pain during swimming. MRI was performed in an effort to differentiate between a solid tumor and a rotator cuff tear. The images showed no rotator cuff injury, but instead a 40 × 40 mm polycystic tumor with solid aspects of the deltoid muscle, which was possibly malignant (Figure 1). Due to the young age of the patient, the suspicion of a malignant tumor was raised and a fine needle biopsy was performed. Histological examination revealed a cystic process with no evidence of malignant cells (Figure 2). The treatment of choice was total surgical excision (Figure 3) with consequent histological analysis of the tissue mass. The two potential diagnoses were intramuscular myxoma and low‐grade fibromyxoid sarcoma. The diagnosis of a benign intramuscular myxoma could be established by excluding the S‐100 staining and rearrangement of the FUS gene, which is diagnostic for fibromyxoid sarcoma. The examination, performed with LSI FUS (16p11) Dual‐Color Break Apart Rearrangement Probe (Figure 4), showed no translocations of the FUS gene and S‐100 staining was also negative, meaning low‐grade fibromyxoid sarcoma was excluded. Therefore, the diagnosis of intramuscular myxoma of the deltoid muscle was reached. Postoperatively, the patient was free of symptoms and could continue swimming without restrictions within a few weeks. No deltoid muscle atrophy was detected postoperatively. A 1‐year follow‐up showed no recurrence.

## 3 DISCUSSION

Intramuscular myxoma is a somatic mosaic disorder occurring as a sporadic isolated lesion, although it can be part of Mazabraud's syndrome (fibrous dysplasia with multiple IM’s) or McCune‐Albright syndrome (fibrous dysplasia, café au lait macules, and endocrine disorders), which are caused by mutations in the GNAS gene.^2^ The case we presented had a single myxoma with no other related pathology. Usually, IM occurs in the fifth to seventh decades of life and incidence has a female predominance. If symptomatic, surgical treatment is recommended. Local recurrence is rare, unless the myxoma is not fully excised or the patient has one of the previously described syndromes.

Histologically, IM is composed of bland spindle‐ and stellate‐shaped cells in an abundant myxoid stroma. Some IM may have areas of hypercellularity and hypervascularity. In this case, a differential diagnosis between myxoid liposarcoma, low‐grade myxofibrosarcoma, and low‐grade fibromyxoid sarcoma should be taken into consideration. Immunohistochemically, IM typically stains negatively for S‐100 protein, whereas myxolipomas and liposarcomas stain positive.^4^

Soft tissue sarcomas (STS) are a heterogeneous group of rare malignant mesenchymal tumors with incidence of two to three cases per 100 000.^5^ Although sarcomas can occur throughout the body, 60% of STS in adults occur in the limbs (15% in the upper extremity and 45% in the lower extremity). Due to the rarity and diversity of these tumors, it is not surprising that excisions are often performed without preoperative suspicion of malignancy and adequate preoperative diagnostic and staging workup.^6^ Tumors of the upper extremity are twice as likely to undergo unplanned excision, probably because of their smaller size and more superficial location.^7^ That is why when a suspicion of a solid tumor is raised, a fine needle biopsy should be performed.

Magnetic resonance imaging is the most sensitive imaging option diagnosing IM. It reveals a well‐defined ovoid mass displaying low signal intensity on T1‐weighted images and high signal intensity on T2‐weighted images. A peripheral and patchy enhancement of the signal after gadolinium injection may be observed. A surrounding fat rim on an MRI is suggestive of IM.^8^ These features are strongly suggestive of intramuscular myxoma.

In this context, we emphasize the role of the radiologist and the pathologist within the differential diagnosis of IM, in order to avoid inappropriate treatments.

## 4 CONCLUSION

MRI is the most sensitive examination for diagnosing tissue injury and tumors. Core needle biopsy may be very helpful for differential diagnosis between benign or malign tumor prior to treatment. Surgical excision is the treatment of choice, but the diagnosis is defined by the histological findings. In the differential diagnosis, reactive lesions and low‐grade myxoid sarcomas should be kept in mind when planning the treatment. Surgical excision has shown to be curative in the vast majority of cases, with minimal recurrence rates.

## CONFLICT OF INTEREST

None declared.

## AUTHOR CONTRIBUTIONS

SA and STM: had the idea for this case report; SA: wrote the first draft of the manuscript; AT, STM, BE, and AM: revised the final manuscript. All authors have read and approved the manuscript.
